# Zoonotic Pathogens in *Ixodes ricinus* from an Urban Environment in Northern Slovakia

**DOI:** 10.3390/pathogens15030292

**Published:** 2026-03-06

**Authors:** Zuzana Cellengová, Blažena Hajdová, Andrea Schreiberová, Patrícia Petroušková, Maroš Kostičák, Alica Kočišová

**Affiliations:** Department of Epizootiology, Parasitology and Protection of One Health, University of Veterinary Medicine and Pharmacy in Košice, Komenského 73, 041 81 Košice, Slovakia; zuzana.cellengova@student.uvlf.sk (Z.C.); blazena.vargova@uvlf.sk (B.H.); andrea.schreiberova@uvlf.sk (A.S.); patricia.petrouskova@uvlf.sk (P.P.); maros.kosticak@student.uvlf.sk (M.K.)

**Keywords:** *Ixodes ricinus*, tick-borne pathogens, Slovakia

## Abstract

*Ixodes ricinus* is the most common and, epidemiologically speaking, the most important tick species in Slovakia, transmitting a wide range of zoonotic pathogens. The goal of the present study was to monitor selected tick-borne infectious agents in an urban environment in northern Slovakia where the conditions for their occurrence and survival are typically unfavourable. Ticks were collected by the flagging method during the period from March to November 2024 in the city of Žilina in five urban locations characterized by high human activity and suitable conditions for tick–host interactions. A total of 264 ticks of *Ixodes ricinus* were collected (67 females, 85 males, and 112 nymphs). A molecular analysis confirmed the presence of *Borrelia* spp. in 34.5% of samples, while the most frequently detected species was *Borrelia afzelii*. The other detected species included zoonotic piroplasms *Babesia microti* and *Babesia venatorum* (1.5%), as well as the bacteria *Anaplasma* spp. (2.65%) and *Rickettsia* spp. (0.4%). In four ticks (1.5%), the presence of coinfection caused by multiple pathogens was detected. These results confirm that urban ecosystems located in the northern regions of Slovakia also provide significant reservoirs of zoonotic pathogens and impose a potential risk for public health.

## 1. Introduction

Climate changes, global warming, and anthropogenic activities have a significant impact on tick ecology. These factors contribute to the growth of tick populations in Europe and their expansion to regions located further north, urban and suburban locations, as well as regions located at higher altitudes [1,2,3].

*Ixodes ricinus* is the most common and epidemiologically the most important species in Slovakia [4,5]. Those ticks are also vectors of a wide range of viral, bacterial, and protozoan pathogens that are important for human and veterinary health [6]. This species is a three-host species, while all of its stages parasitise on a wide range of hosts, including humans [6,7].

Over recent decades, the prevalence of *I. ricinus* has been increasing, even in urban ecosystems. Ticks adapt to new microclimatic conditions, and for their development, they use hosts that live near human dwellings, as has been confirmed in multiple studies conducted in Slovakia [3,8,9,10,11]. *Ixodes ricinus* prefers a cool, humid microclimate, showing higher activity levels when humidity is around 90% [12]. Rainy climates favor their spread, development and reproduction, and they adopt an endophilic lifestyle in summer with high temperatures [13]. *Ixodes ricinus* prefers deciduous and mixed forests, as well as shrub vegetation, while also displaying high ecological plasticity, allowing it to survive in suboptimal climatic conditions and colonize high-altitude environments, reaching altitudes of up to 1800 m above sea level. These adaptive traits, combined with changes in land use and biotope transformation, influence the availability of tick habitat and increase the likelihood of host-tick-pathogen interactions, thus increasing the risk of transmission of tick-borne pathogens [14]. Furthermore, *I. ricinus* shows low host specificity, parasitizing a wide range of vertebrates, including mammals (including humans) and birds [13].

*Ixodes ricinus* is regarded as an important vector of numerous zoonotic pathogens, including tick-borne encephalitis virus (TBEV), spirochetes of the *Borrelia burgdorferi* sensu lato complex (Bbsl), *Anaplasma phagocytophilum*, *Borrelia miyamotoi*, *Coxiella burnetii*, *Neoehrlichia mikurensis*, *Francisella tularensis*, and the protozoa *Babesia microti* and *Babesia venatorum* [9]. Despite these well-known facts, data on the occurrence of zoonotic pathogens in the northern regions of Slovakia remain limited.

The city of Žilina is located in northern Slovakia, a region with a relatively cold climate and an average annual temperature of approximately 8 °C. It was therefore assumed that the local conditions for the occurrence of *I. ricinus* were not optimal. Due to the absence of previous molecular studies conducted in this city, the present study was designed with an aim to examine the occurrence and species composition of ticks and selected tick-borne pathogens in that region.

Bbsl complex, the causative agents of Lyme disease, are the bacteria most frequently transmitted by ticks. The geographical distribution of Lyme disease in Europe is correlated with the presence of *I. ricinus* [15,16,17,18,19,20]. Currently, the Bbsl complex includes 28 genospecies, 7 of which are “Candidatus” [21,22,23,24], 9 of which are pathogenic for humans. In North America, *Borrelia burgdorferi* sensu stricto (Bbss) prevails [25] and *Borrelia mayonii*, which unlike the other Bbsl is spirochaetaemic [26].

In Europe, the following genospecies of the Bbsl complex have been identified: *B. afzelii* [27], Bbss [25], *B. garinii* and *B. bavariensis* [28], *B. lusitaniae* [29], *B. valaisiana* [30], *B. bissettii* [31], *B. spielmanii* [32], *B. finlandensis* [33] and *B. turdi* [34]. *B. garinii* is most prevalent in Central Europe, whereas *B. afzelii* predominates in Northern and Southern Europe [35]. The pathogenicity of *B. finlandensis* [33] and *B. turdi* [34] for humans is not currently documented.

Spotted fever group (SFG) rickettsiae are an important group of tick-borne pathogens ranking among the oldest known tick-borne zoonoses [9]. The prevalence of zoonotic rickettsiae in European ticks has been examined in multiple studies [36]. In Europe, these pathogens manifest in a wide range of clinical symptoms and cause diseases ranging from mild febrile conditions to various severe infections. In Slovakia, the most frequently detected species in the *I. ricinus* population is *Rickettsia helvetica*, followed by *Rickettsia monacensis* [11,37,38].

*Anaplasma phagocytophilum* is a Gram-negative obligate intracellular bacterium [39] that infects neutrophil granulocytes; it is a causative agent of diseases in ruminants, horses, dogs, and humans (human granulocytic anaplasmosis). Its vector in Europe is *I. ricinus* [40,41]. This bacterium exhibits high genetic diversity, while the individual genetic variants are often bound to the particular species of reservoir hosts. Therefore, not all of the strains that circulate in wild ruminants, rodents, and birds impose identically high risks for humans and domestic animals. A positive detection of *Anaplasma* spp. in a tick therefore does not necessarily constitute evidence of the presence of a pathogenic variant of clinical importance [9,42,43,44,45].

As for protozoan pathogens, intra-erythrocytic parasites, *Babesia*, important causative agents of diseases, and their key vectors are hard ticks (Ixodidae) [46,47]. Unlike canine babesiosis, human babesiosis is regarded as a rare disease in Europe. Since the very first case, documented in 1957 in former Yugoslavia [48], approximately 50 cases have been reported on the European continent [49,50,51,52,53]. Although human babesiosis may occur in patients of any age, clinical manifestations have only been observed in patients aged 40–60 years [54,55]. The key risk factors include splenectomy in medical history and immunocompromised conditions, while cases associated with the transmission through blood transfusion have also been observed [56,57,58].

The aforesaid pathogens circulate in enzootic cycles that are affected by urban and wild biotopes, as well as a wide range of reservoir hosts. Understanding their occurrence in different types of environments is a key precondition for the assessment of risks to public health.

The purpose of the present study was to analyse the presence of selected tick-borne pathogens in the population of *I. ricinus* in an urban environment in the city of Žilina, where the climatic conditions are not ideal, and hence contribute to the knowledge of their epidemiological significance in that region. The hypothesis, based on the climate characteristics, was that the local populations of *I. ricinus* have adapted to lower temperatures in this relatively cold climate in northern Slovakia and that such adaptation facilitated their survival and the maintenance of their population.

## 2. Materials and Methods

### 2.1. Selected Locations

The present study was conducted in the city of Žilina (49.223442° N, 18.739328° E), situated at the confluence of the Váh, Kysuca, and Rajčanka rivers at an altitude of 345 m a.s.l. with a surface area of approximately 8000 ha. The town lies in the Žilina basin, surrounded by the Malá Fatra mountains, the Kysuce mountains, the Strážov mountains, the Súľov mountains, and the Javorníky mountains.

Žilina is situated in the temperate zone with a continental climate with an average annual temperature of 7.8 °C and total annual precipitation of ca 1000 mm. The average daily temperature during the period of the peak tick activity was 17 °C.

**Figure 1:**
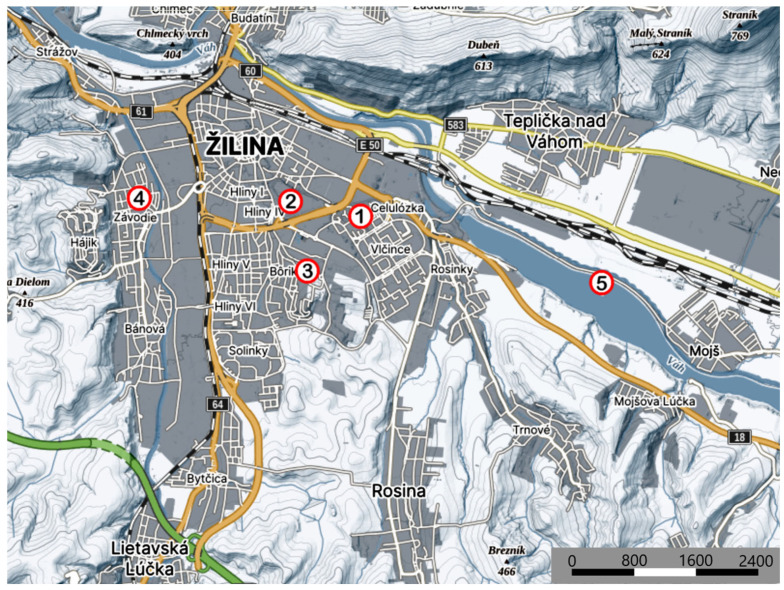
Map of the tick collection locations.

For the purpose of the present investigation, tick samples were collected in three urban and two suburban locations in the city of Žilina with regular mobility of natural hosts and human inhabitants (Table 1). Since the locations differed in vegetation types and ecological conditions, it was possible to assess the effects of various microhabitats on the presence of ticks. All sampling locations are shown on the map in Figure 1.

The location characteristics are as follows:

• Location 1—Urbanised greenery at a housing estate with regularly maintained lawns, bushes and trees, often used for recreational activities. This location typically includes the man-grown vegetation and frequently present pets.
• Location 2—This location includes a hospital area with a high frequency of visitors; the surrounding area consists of meadow grasses, tree alleys, and bushes.
• Location 3—A suburban location situated near a river, with rich vegetation and high air humidity, providing favourable conditions for the presence of ticks. The presence of birds and small mammals may support the circulation of pathogens.
• Location 4—A peripheral recreational zone, partly covered with forests and dense bushes. This location exhibits a relatively high biodiversity of potential hosts, including small mammals and roe deer.
• Location 5—Mutually interconnected greenery bands leading across the urban environment near the Žilina hydroelectric dam, used as migration routes for animals. This type of environment supports the tick populations and facilitates the spread of pathogens in the urbanised area.

### 2.2. Sample Collection

Ticks were collected during the period of March–October 2024, primarily in spring and autumn months when the tick activity reaches its peak values. The collection in the field was carried out on days without precipitation in order to minimise the risk of decreased tick activity and insufficient adhesion of ticks to the wet flag.

Questing ticks were collected from vegetation by the flagging method, using a white cotton flag with a surface area of 1 m^2^. The collection was carried out along the roads, paths, and grassy areas, while the collection areas were divided into 100 m long segments. The segmentation facilitated the identification of a relative tick density (the number of ticks per 100 m^2^). In shrub biotopes, the collection was carried out through static flagging, for a period of 15 min on a single site; this time interval corresponded to a collection surface area of approximately 100 m^2^. The counts observed were used to identify the relative tick density (the number of ticks collected over 15 min).

For the purpose of the present study, only nymphs and adult individuals were collected. Immediately after flagging step, the ticks were stored in the polypropylene test tubes containing 70% ethanol until they were subjected to diagnostic analysis.

After the collection, all ticks were subjected to morphology and sex identification (nymphs, males, females) using a binocular stereomicroscope (Zeiss-Stemi DV-4, Göttingen, Germany) and a diagnostic key [59] . All ticks were identified as *I. ricinus*.

### 2.3. DNA Extraction

DNA extraction was carried out using a commercial DNeasy Blood and Tissue Kit (Qiagen, Hilden, Germany). Prior to the DNA extraction, each tick was taken out of 70% ethanol and allowed to air-dry. Subsequently, each tick was put in a micro test tube and cut into small pieces using a sterile scalpel. Each sample was processed individually. The following steps were carried out in accordance with the protocol provided by the kit manufacturer. The resulting DNA was stored at a temperature of −20 °C for further processing in PCR analysis.

### 2.4. Screening of Pathogens

#### 2.4.1. Laboratory Diagnostics of Borrelias

All samples were analysed for the presence of Borrelia DNA using a PCR assay targeting the intergenic spacer region between the 5S and 23S rRNA genes (5S–23S rRNA IGS) of the Bbsl genogroup, employing the primers IgsF (CGACCTTCTTCGCCTTAAAGC) and IgsR (AGCTCTTATTCGCTGATGGTA) [60]. The reaction cycle consisted of the following steps: 94 °C/4 min; 94 °C/15 s; 57 °C/15 s; 72 °C/20 s; 72 °C/10 min; and the subsequent cooling to 4 °C. The cycle was repeated 35 times.

#### 2.4.2. Laboratory Diagnostics of Rickettsiae

All the samples were analysed for the presence of the DNA of rickettsiae in a PCR reaction with the use of D767f (CGATGGTAGCATTAAAAGCT) and D1390r (CTTGCTTTTCAGCAATATCAC) oligonucleotide primers, which amplify a fragment of the sca4 (surface cell antigen 4) gene with a length of 623 base pairs [61]. This marker has been successfully used in epidemiological surveys of *Rickettsia* spp. in ticks. The reaction cycle consisted of the following steps: 95 °C/30 s; 50 °C/30 s; and 68 °C/90 s. The cycle was repeated 40 times.

#### 2.4.3. Laboratory Diagnostics of Anaplasmae

All the samples were analysed for the presence of DNA of anaplasmae by applying the nested PCR method targeted to the 16S rRNA gene, which is a universal gene for the entire genus. Primers used in the first reaction were ACn-16S-F1 (5′-CACATGCAAGTCGAACGGATTATTC-3′) and ACn-16S-R1 (5′-TTCCGTTAAGAAGGATCTAATCTCC-3′); they amplified a fragment with an expected length of 932 base pairs. The second PCR reaction was carried out with ACn-16S-F2 (5′-AACGGATTATTCTTTATAGCTTGCT-3′) and ACn-16S-R2 (5′-GGCAGTATTAAAAGCAGCTCCAGG-3′) primers, while the length of the target product was 546 base pairs [62]. Reaction conditions in the first PCR reaction were as follows: 94 °C/2 min; 94 °C/30 s; 55 °C/30 s; 72 °C/1 min; 72 °C/5 min; and the subsequent cooling to 4 °C. In the first reaction, the cycle was repeated 40 times, whereas in the second reaction, there were 30 cycles.

#### 2.4.4. Laboratory Diagnostics of Babesia

All the samples were analysed for the presence of DNA of *babesia* by conducting a PCR reaction with the use of BN (TAGTTTATGGTTAGGACTACG) and BJ (GTCTTGTAATTGGAATGATGG) oligonucleotide primers, which amplify a fragment of the 18 s rRNA gene with a length of 450 base pairs [63]. The reaction cycle consisted of the following steps: 94 °C/30 s; 54 °C/30 s; and 72 °C/40 s; and the cycle was repeated 40 times.

For the purpose of confirming the correctness of the reactions, all resulting PCR products were visualised on 1% agarose gel at a voltage of 5 V/cm, immersed in 1× TAE (Tris–acetate–EDTA) buffer. The visualisation of the results in agarose gel was carried out using the GoodView Nucleic Acid Stain (Beijing SBS Genetech Co., Ltd., Beijing, China). Products were then visualized under the UV light.

Positive PCR products of *Borrelia*, *Babesia*, *Anaplasma*, and *Rickettsia* were sent to the SEQme commercial laboratory (Dobříš, Czech Republic) for purification and bidirectional sequencing using the same primers. The sequencing was carried out in the aforementioned laboratory by applying the Sanger sequencing method. The obtained sequences were subsequently analysed and edited in the MEGA X environment, version 10.1.5 (set no. 10191107) [64]. Nucleotide sequences were compiled using the Gene Tool Lite software, version 1.0 (BioTools Inc., Jupiter, FL, USA). The compiled sequences were compared to the sequences deposited in the GenBank database, with the use of the BLAST (Basic Local Alignment Search Tool, version: BLAST+ 2.17.0) nucleotide algorithm in the National Centre for Biotechnology Information (NCBI). The sequences obtained in the study were deposited in the GenBank database under unique accession numbers.

## 3. Results

We collected 264 *Ixodes ricinus* ticks during the study period. The sample included 67 females (25.38%), 85 males (32.19%), and 112 nymphs (42.42%). We collected the highest numbers by flagging in April (*n* = 78; 29.55%) and in May (*n* = 65; 24.62%), corresponding to the spring peak of tick activity. The second important peak occurred in September (*n* = 35; 13.26%), reflecting the bimodal seasonal activity pattern of this species.

**Figure 2:**
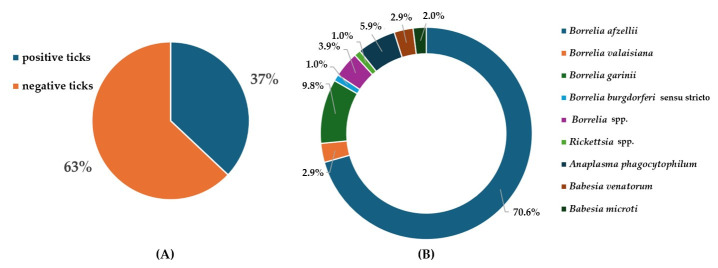
(**A**) Total percentages of positive and negative *Ixodes ricinus* ticks, analysed for tick-borne pathogens. (**B**) Relative proportion of detected pathogen species among all detected infections (*n* = 102). Percentages were calculated using the total number of detected infections as the denominator; therefore, ticks with co-infections contributed more than one detection.

In 34.5% of ticks, the infection caused by borrelia was confirmed; 0.4% of samples were confirmed to be infected by rickettsiae; 2.65% of ticks were confirmed to be infected by anaplasmae; and in 1.9% of ticks, infection caused by babesia was confirmed. Out of the total number of ticks, 4 individuals (1.5%) were confirmed to be infected with more than one pathogen. In two cases, *B. afzelii* and *B. microti* (a female and a nymph) were present concurrently; in one case (a male), a coinfection caused by *B. afzelii* and *Rickettsia* spp. was detected; and there was one case (a nymph) of a coinfection caused by *B. afzelii* and *Anaplasma phagocytophilum*. A total of 102 pathogen infections were detected. Figure 2A presents the total positivity for *Ixodes ricinus*, and Figure 2B shows the relative proportion of detected pathogen species, including co-infections. Percentages were calculated using the total number of detected infections as the denominator; therefore, co-infected ticks contributed more than one detection.

### 3.1. Results of the Screening of Pathogens

#### 3.1.1. Screening of Borrelia

DNA of *Borrelia* was detected in 91 out of 264 analysed ticks (34.5%). The results of molecular identification of *Borrelia* spp., including life stages and coinfections, are shown in Table 2. By sequencing the amplified fragments, the presence of *B. afzelii*, *B. valaisiana*, *B. garinii*, and Bbss was confirmed. The most frequently detected genotype was *Borrelia afzelii*, which was found in all of its developmental stages (*n* = 73; 27.7%), followed by *B. garinii*, which was also present in all developmental stages (*n* = 10; 3.8%). *B. valaisiana* was detected in a single male and two females (*n* = 3; 1.1%), while Bbss was found in a single female (*n* = 1; 0.4%). In four cases (1.5%), male individuals were confirmed to be positive for *Borrelia* spp. However, the species identification, conducted by sequence analysis, did not allow species level identification. The dominance of the *B. afzelii* genotype indicated a close association with rodent populations as the main reservoirs in the urban environment of Žilina [65]. The highest number of positive ticks originated in Location 5 (45/91; 49.5%).

Detailed information on the molecular identification, life stages, localities, and detected coinfections of Borrelia-positive ticks is presented in Table 2.

#### 3.1.2. Screening of Rickettsia

The DNA of *Rickettsia* was detected in a single male (0.4%)—the presence of *Rickettsia* spp. was confirmed. Although the prevalence was low, this finding indicated that rickettsiae circulate in the urban environment. The positive tick originated in Location 4.

The positive sample was subjected to sequencing; however, the sequence quality was insufficient for reliable species identification and was therefore reported as *Rickettsia* spp.

#### 3.1.3. Screening of Anaplasma

The DNA of *Anaplasma* was detected in 6 out of 264 ticks (2.27%). Sequencing confirmed all positive samples as *Anaplasma phagocytophilum*. The positive samples were detected in various developmental stages of *I. ricinus*. Positivity was observed in 3 males (*n* = 3/85; 3.53%), 1 female (*n* = 1/67; 1.49%), and 2 nymphs (*n* = 2/112; 1.79%). The majority of positive ticks was found in Location 4 (4 ticks), while 2 positive samples were taken from Location 5. Detailed information on the molecular identification, life stages, localities, and detected coinfections of Anaplasma-positive ticks is presented in Table 3.

#### 3.1.4. Screening of Babesia

The DNA of *Babesia* was detected in 5 out of 264 analysed ticks (1.9%). Details on the molecular identification, life stages, localities, and detected coinfections of *Babesia*-positive ticks are summarised in Table 3. By sequencing of the amplified fragments of the 18S rRNA gene, two zoonotic species were identified—*B. microti* (*n* = 3; 1.14%) and *B. venatorum* (*n* = 2; 0.76%). Sequencing showed 100% identity with reference sequences deposited in GenBank. *Babesia venatorum* was confirmed in females only (2/67; 3.0%), and *B. microti* was present in one female individual (1/67; 1.5%) and in nymphs (2/112; 1.8%). Both species have zoonotic potential and their occurrence in the urban environment imposes a risk to the human population. Because *B. microti* is not a zoonotic pathogen in all genotypes, the results observed in the present study were compared to the GenBank database with the result of a 100% match with the zoonotic isolate originating in Jena, Germany [66]. Positive samples were collected in Location 2 (1 tick), Location 4 (1 tick), and Location 5 (3 ticks).

Detailed information on the molecular identification, life stages, localities, and detected coinfections of Babesia-positive ticks is presented in Table 4.

**Table 4:** Molecular identification of *Babesia* spp. detected in *I. ricinus*.

Sample No.	Locality	Life Stage	Molecular Identification	GenBank	Highest Sequence Identity with GenBank		Coinfection with Another Pathogen
					Identity	Accession Number	
2IRF30	N°2	female	*Babesia venatorum*	PX931523	100%	KJ465868.1	
4IRF77	N°4	female	*Babesia venatorum*	PX931524	100%	KJ663730.1	
5IRF181	N°5	female	*Babesia microti*	PX931525	100%	EF413181.1	*Borrelia (Borreliella) afzelii*
5IRN217	N°5	nymph	*Babesia microti*	PX931526	100%	EF413181.1	
5IRN242	N°5	nymph	*Babesia microti*	PX931527	100%	EF413181.1	*Borrelia (Borreliella) afzelii*

## 4. Discussion

The presence of ticks is usually associated with rural and forest environments because they prefer biotopes with dense vegetation and the presence of natural hosts, namely wild animals. However, research indicates that ticks have successfully adapted to urban environments too, and hence became important sources of infection that affect humans [11,67,68]. Despite the fact that the research conducted over the last decade has documented a spread of *Ixodes ricinus* to urban parks, gardens, and suburban biotopes [3,8,69], no molecular studies have been conducted directly in the city of Žilina to analyse the species composition of the tick population as well as the tick-borne pathogens.

In the present study, 264 *I. ricinus* individuals were collected. The highest prevalence was observed in spring months, which is a finding that is consistent with a typical seasonal activity peak of this species in the temperate climate zone and corresponds to the results of other studies conducted in Slovakia and neighbouring countries [3,4]. These findings confirm that *I. ricinus* is capable of long-term survival and maintains stable populations in urban environments in northern Slovakia despite the insufficiently favourable climate conditions. This outcome has also been facilitated by suitable microclimate conditions that have been created in the urban green zones, such as parks, riparian vegetation, and bio-corridors, as they provide sufficient humidity and sites suitable for hiding [9,11].

The detection of multiple pathogens in the analysed samples confirmed that urban tick populations constitute an important element from an epidemiological point of view. Therefore, the species composition and the prevalence of individual pathogens were also analysed.

The analysis included nymphs and adult individuals only since the vertical transmission of borrelia (from a female to a larva) only rarely occurs; this means that the unfed larvae become infected only in exceptional cases [32]. An infected nymph receives borrelia while a larva is feeding on a reservoir host. European birds are the reservoirs of avian genotypes (*B. garinii*, *B. valaisiana*, and *B. turdi*), while mammals are the hosts for genotypes from different ecological groups (*B. afzelii*, Bbss, and *B. spielmanii*) [65]. The identification of a specific genotype in an infected nymph therefore facilitates an indirect identification of the type of host on which the larva had been feeding. Adult ticks may be infected by genotypes originating in both mammals and birds since they have ingested blood twice during their life cycle, which is not the case for larvae and nymphs [65].

The Bbsl complex has been studied in a large number of epidemiological studies conducted in Europe [70,71,72]. The average prevalence of Bbsl in ticks in Europe is approximately 15% [72], while the highest values have been reported from central Europe and the southern parts of Scandinavia [70]. The prevalence of *Borrelia* spp., observed in the present study, amounted to 34.5%—a value that is higher than the majority of data on the Slovakian urban and suburban biotopes. According to the available literature, the prevalence of *Borrelia* spp. in ticks from vegetation (questing ticks) in Slovakia significantly varies in different regions and biotope types—from 4.4% in suburban forests in northern Slovakia [3] to more than 50% in certain locations in eastern Slovakia [73]. In urban biotopes, the prevalence values are usually lower than those identified in natural forest ecosystems, as has been confirmed in several studies conducted in Slovakia [3,71,73,74,75]. Nevertheless, there are certain urban locations where the values observed were similar to those identified in natural biotopes—for instance, the urban park in Malacky where the prevalence amounted to approximately 20% [20,76]. The value identified in the present study (34.5%) is higher than the values typically observed in urban parks, albeit still within the range observed for suburban biotopes. Similar long-term monitoring studies in European urban environments have demonstrated that urban populations of *Ixodes ricinus* may maintain stable circulation of multiple *Borrelia* genospecies and reach comparable prevalence levels [77]. This study has also shown that borreliae were present in ticks in co-infection with other pathogen species. In addition, co-occurrence of different *Borrelia* genospecies within a single tick has been widely documented in the literature [77,78]. Since Borrelia genotypes identification in the present study was based on Sanger sequencing, which predominantly detects the dominant sequence within a sample, the presence of minor co-occurring genospecies cannot be completely excluded. Consequently, the actual diversity of Borrelia spp. in the studied tick population may be slightly underestimated.

Several rickettsia species, primarily *R. helvetica* and *R. monacensis*, are regularly detected in *I. ricinus* populations that are present in various European biotopes, while their prevalence ranges from less than 1% to more than 30%, depending on the country and environmental conditions [36,37,38,79,80,81,82,83,84,85,86,87,88]. Lower values (0.5–8%) are usually observed in central Europe, for example in Germany and Italy [83,88], while significantly higher values (˃20%) have been reported from countries located farther south, such as Spain and Turkey [84,85]. In Slovakia, studies conducted in the north-west part of the country reported prevalence values of 6–9% [11,37].

In the ticks analysed in the present study, *Rickettsia* spp. was confirmed in a single tick reach comparable prevalence levels, representing a prevalence of 0.38% (*n* = 1/264)—a value that is significantly lower than the majority of data reported from Europe, including Slovakia [37]. The positive sample could not be reliably identified to the species level despite sequencing and was therefore conservatively classified as *Rickettsia* spp. Additionally, PCR assays targeting the sca4 gene may show variable sensitivity depending on the *Rickettsia* species present, which may also contribute to differences in reported prevalence. Despite the low prevalence, the occurrence of *Rickettsia* spp. in the urban ecosystem is relevant, since some of the species in that group are regarded as potentially pathogenic for humans.

The prevalence of *A. phagocytophilum* in ticks depends on the presence of suitable reservoir hosts. In Europe, the prevalence of *I. ricinus* ranges from 0.4 to 66.7% [89]. In the present study, DNA of *A. phagocytophilum* was detected in 2.27% of ticks (three males, one female, and two nymphs). The positive individuals originated in Locations 4 and 5 only; this may indicate uneven spatial distribution of the pathogen and the reservoir hosts in the urban environment. Compared to the results of the previous studies conducted in Slovakia, in which the observed prevalence of *A. phagocytophilum* in urban and suburban biotopes ranged from 0.6 to 30% [3,10,42,74,75,90], the prevalence value observed in this study ranks among the lower values.

The most frequently reported causative agent of human babesiosis in Europe is *Babesia divergens*; however, various case studies increasingly report that this disease is caused *B. venatorum* and *B. microti*—the species detected in the present study [57,66,91]. DNA of *Babesia* spp. was detected in 5 out of 264 ticks (1.9%), while two zoonotically significant species were identified—*B. microti* (*n* = 3; 1.14%) and *B. venatorum* (*n* = 2; 0.76%). *B. microti* genotypes exhibited a 100% match with the zoonotic isolate originating in Germany [66]. This finding confirms their importance with regard to human health. The positive individuals originated in Locations 2, 4, and 5. This indicates that the life cycle of those parasites may also take place in an urban biotope. In Europe, the infections caused by *Babesia* spp. are typically observed with only a low prevalence, despite the fact that the published values range from 9 to 20% [63,80,92,93,94,95,96,97,98,99,100,101,102,103,104,105]. Data available from serology studies indicate that the real prevalence of babesiosis in Europe may be much higher than the values indicated by clinical data. That may be related to the fact that the course of the disease is often asymptomatic and accompanied by non-specific symptoms that lead to underdiagnosis or incorrect diagnosis [106,107]. At present, human babesiosis is regarded as an emerging disease; however, the global number of reported cases has been increasing over the last years [46,50,51,53]. Therefore, the findings of this study indicate the need for further systematic monitoring.

The present findings indicate that the urban and peri-urban environments of Žilina provide suitable conditions for the occurrence of *I. ricinus* and the circulation of zoonotic pathogens, even under less favorable climatic conditions. Several positive ticks were collected in locations characterized by mixed urban and semi-natural habitats, which may support tick survival and host availability. However, long-term multi-year monitoring would be necessary to confirm the existence of stable, self-sustaining tick populations in strictly urbanized areas. The prevalence of *Borrelia* spp., *Anaplasma phagocytophilum*, *Rickettsia* spp., and piroplasms (*B. microti* and *B. venatorum*) indicates the circulation of tick-borne pathogens in the urban environment of Žilina. This study presents the first molecular evidence of the presence of these pathogens in this city.

## 5. Conclusions

The results of this study clearly indicate that an urban environment should be regarded as an important and often underestimated area, providing conditions favourable for the spread of tick-borne pathogens. The results confirm that even in the less favourable climatic conditions of northern Slovakia, urban environments may support stable populations of *I. ricinus* and maintain the circulation of multiple zoonotic pathogens. In the town of Žilina, a high prevalence of *Borrelia* spp. was detected (34.5%), as was the presence of other clinically significant pathogens—*Anaplasma* spp., *Rickettsia* spp., and the zoonotic species of babesia *B. microti* and *B. venatorum*. This imposes a real exposure risk for the inhabitants of those regions and their domestic and companion animals.

The obtained results provide the first ever molecular confirmation of the presence of the studied pathogens in Žilina; they also confirm the importance of conducting further research into the dynamics of ticks and the pathogens they transmit in urban ecosystems, including the role of reservoir hosts and anthropogenic factors.

## Figures and Tables

**Table 1:** Location characteristics and the amounts of collected ticks.

Location No.	Location Name	GPS Coordinates	Urban/Suburban	Sample (*n*)	n/100 m^2^
1	Ľudovít Fulla Square	49.21573937138863, 18.761430279826975	U	6	0.06
2	Nemocničná Street	49.216119257499884, 18.752342711580464	U	30	0.3
3	Rajčanka	49.20844738562995, 18.72316370267406	U	25	0.25
4	Hájovňa	49.208168062445715, 18.75275277132755	S	100	1
5	Bio-corridor	49.206477461863514, 18.809388182210647	S	101	1.01

**Table 2:** Molecular identification of *Borrelia* spp. detected in *I. Ricinus*.

Sample No.	Locality	Life Stage	Molecular Identification	GenBank	Highest Sequence Identity with GenBank		Coinfection with Another Pathogen
					Identity	Accession Number	
1IRF1	N°1	female	*Borrelia (Borreliella) afzelii*	PX961465	100%	CP075204.1	
1IRM2	N°1	male	*Borrelia (Borreliella) garinii*	PX961466	100%	CP028861.1	
1IRM3	N°1	male	*Borrelia (Borreliella) garinii*	PX961467	100%	MG356956.1	
2IRM7	N°2	male	*Borrelia (Borreliella) afzelii*	PX961468	100%	OP559184.1	
2IRM14	N°2	male	*Borrelia (Borreliella) garinii*	PX961469	100%	MG356954.1	
2IRF22	N°2	female	*Borrelia (Borreliella) afzelii*	PX961470	100%	OP559180.1	
2IRF25	N°2	female	*Borrelia (Borreliella) garinii*	PX961471	100%	MG356956.1	
2IRF26	N°2	female	*Borrelia (Borreliella) afzelii*	PX961472	100%	OP559180.1	
2IRF28	N°2	female	*Borrelia (Borreliella) burgdorferi*	PX961473	100%	OP559187.1	
2IRF34	N°2	female	*Borrelia (Borreliella) afzelii*	PX961474	100%	OP559180.1	
2IRF35	N°2	female	*Borrelia (Borreliella) afzelii*	PX961475	100%	OP559185.1	
3IRF39	N°3	female	*Borrelia (Borreliella) garinii*	PX961476	100%	MK256774.1	
3IRF41	N°3	female	*Borrelia (Borreliella) valaisiana*	PX961477	100%	PV925661.1	
3IRF45	N°3	female	*Borrelia (Borreliella) garinii*	PX961478	100%	MG356954.1	
3IRF49	N°3	female	*Borrelia (Borreliella) garinii*	PX961479	100%	CP075422.1	
3IRM52	N°3	male	*Borrelia (Borreliella) afzelii*	PX961480	99.64%	CP075206.1	
3IRM53	N°3	male	*Borrelia (Borreliella) garinii*	PX961481	100%	CP075413.1	
3IRM58	N°3	male	*Borrelia (Borreliella) valaisiana*	PX961482	100%	MW489021.1	
4IRF65	N°4	female	*Borrelia (Borreliella) afzelii*	PX961483	100%	OP559180.1	
4IRN69	N°4	nymph	*Borrelia (Borreliella) afzelii*	PX961484	99.65%	CP075442.1	
4IRM73	N°4	male	*Borrelia (Borreliella) afzelii*	PX961485	99.65%	CP075446.1	
4IRF74	N°4	female	*Borrelia (Borreliella) afzelii*	PX961486	99.65%	CP075442.1	
4IRM83	N°4	male	*Borrelia (Borreliella) garinii*	PX961487	100%	OL848233.1	
4IRM87	N°4	male	*Borrelia (Borreliella) afzelii*	PX961488	99.28%	CP075446.1	
4IRM90	N°4	male	*Borrelia (Borreliella) afzelii*	PX961489	99.64%	CP075206.1	
4IRF98	N°4	female	*Borrelia (Borreliella) afzelii*	PX961490	99.30%	CP075206.1	
4IRF102	N°4	female	*Borrelia (Borreliella) afzelii*	PX961491	100%	CP075446.1	
4IRM103	N°4	male	*Borrelia (Borreliella) afzelii*	PX961492	100%	CP009212.1	
4IRM106	N°4	male	*Borrelia (Borreliella) afzelii*	PX961493	99.24%	OP559184.1	
4IRM115	N°4	male	*Borrelia (Borreliella) afzelii*	PX961494	99.62%	PP874245.1	*Rickettsia* spp.
4IRN117	N°4	nymph	*Borrelia (Borreliella) afzelii*	PX961495	100%	OP559182.1	
4IRN121	N°4	nymph	*Borrelia (Borreliella) garinii*	PX961496	99.63%	MG356951.1	
4IRN131	N°4	nymph	*Borrelia (Borreliella) afzelii*	PX961497	100%	CP009212.1	
4IRM138	N°4	male	*Borrelia (Borreliella) afzelii*	PX961498	99.62%	OP559180.1	
4IRM142	N°4	male	*Borrelia (Borreliella) afzelii*	PX961499	100%	CP075206.1	
4IRF143	N°4	female	*Borrelia (Borreliella) afzelii*	PX961500	99.62%	OP559180.1	
4IRF144	N°4	female	*Borrelia (Borreliella) afzelii*	PX961501	100%	OP559183.1	*Anaplasmaphagocytophilum*
4IRM145	N°4	male	*Borrelia (Borreliella) afzelii*	PX961502	100%	OP559184.1	
4IRM146	N°4	male	*Borrelia (Borreliella) valaisiana*	PX961503	100%	AF497987.1	
4IRN150	N°4	nymph	*Borrelia (Borreliella) afzelii*	PX961504	99.62%	OP559182.1	
4IRN151	N°4	nymph	*Borrelia (Borreliella) afzelii*	PX961505	100%	OP559184.1	
4IRN155	N°4	nymph	*Borrelia (Borreliella) afzelii*	PX961506	100%	OP559180.1	
4IRN157	N°4	nymph	*Borrelia (Borreliella) afzelii*	PX961507	100%	OP559183.1	
4IRN160	N°4	nymph	*Borrelia (Borreliella) afzelii*	PX961508	99.59%	AB178356.1	
4IRN161	N°4	nymph	*Borrelia (Borreliella) afzelii*	PX961509	100%	OP559183.1	
5IRN163	N°5	nymph	*Borrelia (Borreliella) afzelii*	PX961510	99.59%	EF488988.1	*Anaplasmaphagocytophilum*
5IRN164	N°5	nymph	*Borrelia (Borreliella) afzelii*	PX961511	100%	CP075442.1	
5IRF169	N°5	female	*Borrelia (Borreliella) afzelii*	PX961512	100%	CP075248.1	
5IRF171	N°5	female	*Borrelia (Borreliella) afzelii*	PX961513	100%	AY772041.1	
5IRF173	N°5	female	*Borrelia (Borreliella) afzelii*	PX961514	98.87%	CP075446.1	
5IRF175	N°5	female	*Borrelia (Borreliella) afzelii*	PX961515	100%	PV925630.1	
5IRF177	N°5	female	*Borrelia (Borreliella) afzelii*	PX961516	100%	OP559181.1	
5IRF178	N°5	female	*Borrelia (Borreliella) afzelii*	PX961517	100%	CP075446.1	
5IRF179	N°5	female	*Borrelia (Borreliella) afzelii*	PX961518	100%	KX418638.1	
5IRF181	N°5	female	*Borrelia (Borreliella) afzelii*	PX961519	99.64%	CP075206.1	*Babesiamicroti*
5IRF182	N°5	female	*Borrelia (Borreliella) afzelii*	PX961520	100%	CP075446.1	
5IRF183	N°5	female	*Borrelia (Borreliella) afzelii*	PX961521	100%	CP009212.1	
5IRM187	N°5	male	*Borrelia (Borreliella) afzelii*	PX961522	99.64%	CP075446.1	
5IRM189	N°5	male	*Borrelia (Borreliella) afzelii*	PX961523	100%	CP009212.1	
5IRM191	N°5	male	*Borrelia (Borreliella) afzelii*	PX961524	100%	MW924130.1	
5IRM196	N°5	male	*Borrelia (Borreliella) afzelii*	PX961525	100%	CP075448.1	
5IRM200	N°5	male	*Borrelia (Borreliella) afzelii*	PX961526	100%	CP009212.1	
5IRN205	N°5	nymph	*Borrelia (Borreliella) afzelii*	PX961527	100%	CP075446.1	
5IRN206	N°5	nymph	*Borrelia (Borreliella) afzelii*	PX961528	100%	CP075248.1	
5IRN208	N°5	nymph	*Borrelia (Borreliella) afzelii*	PX961529	100%	CP075442.1	
5IRN210	N°5	nymph	*Borrelia (Borreliella) afzelii*	PX961530	100%	PV925565.1	
5IRN212	N°5	nymph	*Borrelia (Borreliella) afzelii*	PX961531	100%	CP009212.1	
5IRN214	N°5	nymph	*Borrelia (Borreliella) afzelii*	PX961532	99.64%	CP075206.1	
5IRN225	N°5	nymph	*Borrelia (Borreliella) afzelii*	PX961533	99.28%	CP009212.1	
5IRN226	N°5	nymph	*Borrelia (Borreliella) afzelii*	PX961534	100%	CP075446.1	
5IRN227	N°5	nymph	*Borrelia (Borreliella) afzelii*	PX961535	100%	JX909859.1	
5IRN228	N°5	nymph	*Borrelia (Borreliella) afzelii*	PX961536	98.92%	CP075446.1	
5IRN229	N°5	nymph	*Borrelia (Borreliella) afzelii*	PX961537	100%	OP559184.1	
5IRN233	N°5	nymph	*Borrelia (Borreliella) afzelii*	PX961538	98.92%	CP075442.1	
5IRN234	N°5	nymph	*Borrelia (Borreliella) afzelii*	PX961539	100%	CP075446.1	
5IRN238	N°5	nymph	*Borrelia (Borreliella) afzelii*	PX961540	98.92%	CP075446.1	
5IRN240	N°5	nymph	*Borrelia (Borreliella) afzelii*	PX961541	99.64%	CP075442.1	
5IRN241	N°5	nymph	*Borrelia (Borreliella) afzelii*	PX961542	99.64%	CP075206.1	
5IRN242	N°5	nymph	*Borrelia (Borreliella) afzelii*	PX961543	98.92%	CP075446.1	*Babesiamicroti*
5IRN244	N°5	nymph	*Borrelia (Borreliella) afzelii*	PX961544	99.64%	CP075206.1	
5IRN246	N°5	nymph	*Borrelia (Borreliella) afzelii*	PX961545	99.64%	CP075446.1	
5IRN247	N°5	nymph	*Borrelia (Borreliella) afzelii*	PX961546	100%	CP009212.1	
5IRN252	N°5	nymph	*Borrelia (Borreliella) afzelii*	PX961547	98.92%	CP075446.1	
5IRN253	N°5	nymph	*Borrelia (Borreliella) afzelii*	PX961548	99.64%	CP075206.1	
5IRN254	N°5	nymph	*Borrelia (Borreliella) afzelii*	PX961549	100%	CP075446.1	
5IRN259	N°5	nymph	*Borrelia (Borreliella) afzelii*	PX961550	99.28%	CP075446.1	
5IRN260	N°5	nymph	*Borrelia (Borreliella) afzelii*	PX961551	98.92%	CP075446.1	
5IRN263	N°5	nymph	*Borrelia (Borreliella) afzelii*	PX961552	99.64%	CP075206.1	
5IRN264	N°5	nymph	*Borrelia (Borreliella) afzelii*	PX961553	99.64%	CP075446.1	

**Table 3:** Molecular identification of *Anaplasma* spp. detected in *I. ricinus*.

Sample No.	Locality	Life Stage	Molecular Identification	GenBank	Highest Sequence Identity with GenBank		Coinfection with Another Pathogen
					Identity	Accession Number	
4IRM89	N°4	male	*Anaplasma phagocytophilum*		100%	EU839849.1	
4IRM139	N°4	male	*Anaplasma phagocytophilum*		100%	EU839851.1	
4IRF144	N°4	female	*Anaplasma phagocytophilum*		100%	EU839851.1	*Borrelia (Borreliella) afzelii*
4IRN156	N°4	nymph	*Anaplasma phagocytophilum*		99.62%	EU839848.1	
5IRN163	N°5	nymph	*Anaplasma phagocytophilum*		100%	EU839848.1	*Borrelia (Borreliella) afzelii*
5IRM186	N°5	male	*Anaplasma phagocytophilum*		100%	EU839851.1	

## Data Availability

Data presented in this article are available in the manuscript in tables and figures. The respective data can be found in GenBank at https://www.ncbi.nlm.nih.gov/genbank/ under the numbers PX931523-PX931527 and PX961465-PX961553.
